# Highly Reduced Genome of the New Species *Mycobacterium uberis*, the Causative Agent of Nodular Thelitis and Tuberculoid Scrotitis in Livestock and a Close Relative of the Leprosy Bacilli

**DOI:** 10.1128/mSphere.00405-18

**Published:** 2018-10-03

**Authors:** Andrej Benjak, Charlotte Avanzi, Yvonne Benito, Franck Breysse, Christophe Chartier, Maria-Laura Boschiroli, Christine Fourichon, Lorraine Michelet, Didier Pin, Jean-Pierre Flandrois, Pierre Bruyere, Oana Dumitrescu, Stewart T. Cole, Gerard Lina

**Affiliations:** a Global Health Institute, École Polytechnique Fédérale de Lausanne, Lausanne, Switzerland; b Institut des Agent infectieux, Hôpital de la Croix Rousse, Hospices Civils de Lyon, Lyon, France; c CIRI, Centre International de Recherche en Infectiologie, Inserm U1111, Université Lyon 1, Ecole Normale Supérieure de Lyon, CNRS UMR 5308, Lyon, France; d Laboratoire d’Immunologie, Centre de Biologie Sud, Lyon, France; e BIOEPAR, INRA, Oniris, La Chantrerie, Nantes, France; f ANSES Laboratory Affairs Department, Maisons-Alfort, France; g Université de Lyon, VetAgro Sup, UPSP 2016.A104, Interactions Cellules Environnement, Marcy l’Etoile, France; h Université Lyon1, LBBE-CNRS UMR5558, Villeurbanne, France; i Institut Pasteur, Paris, France; Washington University in St. Louis School of Medicine

**Keywords:** *Mycobacterium uberis*, evolutionary biology, genome analysis, granulomatous dermatitis, veterinary pathogens

## Abstract

Nodular thelitis is a chronic enzootic infection affecting dairy cows and goats. The causative agent was recently shown to be related to the leprosy-causing bacilli Mycobacterium leprae and Mycobacterium lepromatosis. In this study, the genome of this pathogen was sequenced and analyzed. Phylogenomic analyses confirmed that the pathogen present in nodular thelitis and tuberculoid scrotitis is a distinct species related to the leprosy bacilli and Mycobacterium haemophilum. Because the pathogen was originally isolated from a bovine udder, it was named “*Mycobacterium uberis*.” The genome of “*M. uberis*” is only 3.12 Mb in length, which represents the smallest mycobacterial genome identified so far but which is close to that of leprosy bacilli in size. The genome contains 1,759 protein-coding genes and 1,081 pseudogenes, indicative of extensive reductive evolution and likely the reason that M. uberis cannot be grown axenically. The pseudogenization and genome reduction in M. uberis seem to have been to some extent independent from the results determined for the genomes of the leprosy bacilli.

**IMPORTANCE** M. uberis is an emerging skin pathogen in dairy animals. Its genome underwent massive reduction and gene decay, leading to a minimal set of genes required for an obligatory intracellular lifestyle, which highly resembles the evolution of the leprosy agents M. leprae and M. lepromatosis. The genomic similarity between M. uberis and the leprosy bacilli can help in identifying key virulence factors of these closely related species or in identifying genes responsible for the distinct differences between thelitis or scrotitis and leprosy with respect to clinical manifestations. Specific DNA markers can now be developed for quick detection of this pathogen.

## Abstract (precis)

M. uberis is an emerging skin pathogen in dairy animals. Its genome underwent massive reduction and gene decay, leading to a minimal set of genes required for an obligatory intracellular lifestyle, which highly resembles the evolution of the leprosy agents M. leprae and M. lepromatosis. The genomic similarity between M. uberis and the leprosy bacilli can help in identifying key virulence factors of these closely related species or in identifying genes responsible for the distinct differences between thelitis or scrotitis and leprosy with respect to clinical manifestations. Specific DNA markers can now be developed for quick detection of this pathogen.

## INTRODUCTION

Nodular thelitis is a chronic, enzootic granulomatous dermatitis associated with acid-fast bacilli. It was originally observed in cows and was first described in France in 1963 (1) and then in Japan (2) and Switzerland (3). A similar disease, nodular tuberculoid scrotitis, was observed in bulls and is suspected to be caused by the same pathogen. The causative agent of the bovine nodular thelitis was recently shown to be related to the leprosy-causing species Mycobacterium leprae and Mycobacterium lepromatosis (4). More recently, the same pathogen was also identified in dairy goats (5). In this study, the draft genome of this pathogen was reconstructed and analyzed to reveal a distinct mycobacterial species and for use for confirmation of its detection in nodular thelitis and tuberculoid scrotitis.

## RESULTS AND DISCUSSION

### Species name and phylogeny.

**FIG 1:**
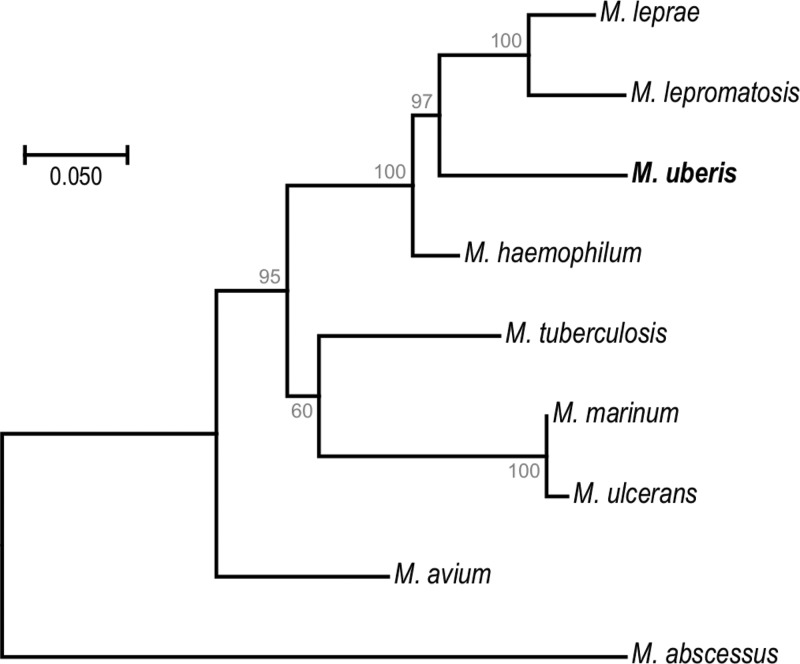
Phylogenetic tree of *Mycobacterium uberis* and selected mycobacterial species. The tree was created in MEGA7 from concatenated amino acid sequences (3,696 positions) of 10 proteins (DnaN, RplI, GrpE, MetG, RplY, PheT, FtsQ, HolA, MiaA, and FtsY) (18) and inferred by using the maximum likelihood method based on the JTT matrix-based model. The tree is drawn to scale, with branch lengths measured at the number of substitutions per site. Bootstrap support values, estimated from 500 replicates, are given below each branch. *Mycobacteroides abscessus* (previously Mycobacterium abscessus) was used as the outgroup.

As shown in Fig. 1, the new species forms a distinct branch lying between M. haemophilum and the most recent common ancestor of M. leprae and Mycobacterium lepromatosis. In view of its initial identification from udder, this species is named Mycobacterium uberis.

### Mycobacterium uberis genome sequence.

DNA was isolated from a skin biopsy of bovine udder with nodular thelitis and subjected to Illumina sequencing. The *de novo* sequence assembly resulted in 3,571 contigs that were larger than 1 kb and showed average coverage of over 10×. Most contigs matched sequences from a variety of bacterial species. On the basis of sequence similarity to M. haemophilum and M. leprae, we retrieved contigs that unmistakably belonged to M. uberis. Care was taken not to exclude any other potential M. uberis sequences by manually checking all the remaining contigs that displayed GC content similar to that of M. uberis and by repeating the analysis with another assembly program (see Text S1 in the supplemental material for details). The draft M. uberis genome assembly consists of 54 contigs with an average length of 58 kb and totaling 3.12 Mb. All of the contigs harbored genes that closely match those of M. haemophilum (85.7% average nucleotide identity) or M. leprae (82.4% average nucleotide identity), with no obvious outlier that would indicate an erroneous assembly with sequences from another bacterial species.

10.1128/mSphere.00405-18.1 TEXT S1 Supplemental Materials and Methods. Download Text S1, PDF file, 0.1 MB. Copyright © 2018 Benjak et al. 2018 Benjak et al. This content is distributed under the terms of the Creative Commons Attribution 4.0 International license.

### Genome downsizing and pseudogene formation.

At a sequence length of 3.12 Mb and containing 1,081 pseudogenes, the genome of M. uberis is as reduced as that of M. leprae (Table 1). The two species share 1,318 functional protein-coding genes, which corresponds to 75% of the total number of protein-coding genes in M. uberis and 82% in M. leprae. Similarly, among the 1,309 pseudogenes in M. leprae, only 212 orthologs were predicted to be functional in M. uberis; conversely, among the 1,081 pseudogenes in M. uberis, only 126 orthologs are predicted to be functional in M. leprae (see Data Set S1 in the supplemental material**)**.

**TABLE 1:** Genomic features of *M. uberis* and close relatives *M. leprae*, *M. lepromatosis*, and *M. haemophilum*

Feature	Value			
	*M. uberis*	*M. leprae*	*M. lepromatosis*	*M. haemophilum*
Genome size (bp)	3,122,721	3,268,212	3,206,741	4,235,765
No. of protein-coding genes	1,759	1,609	1,477	3,749
No. of pseudogenes	1,081	1,309	1,334	225
% GC content (genome)	57.49	57.80	57.89	63.95
% GC content (CDS)^a^	59.58	60.11	60.16	64.35
% GC content (pseudogenes)	55.60	56.45	56.59	64.84
% GC content (intergenic)	53.08	54.17	54.61	60.37

a CDS, coding DNA sequence.

10.1128/mSphere.00405-18.4 DATA SET S1 Mycobacterium uberis Jura genome annotation. Download Data Set S1, XLSX file, 1.8 MB. Copyright © 2018 Benjak et al. 2018 Benjak et al. This content is distributed under the terms of the Creative Commons Attribution 4.0 International license.

The pseudogene content of M. uberis differs from that of M. leprae. For example, 465 pseudogenes in M. uberis do not have an ortholog in M. leprae, and 607 pseudogenes in M. leprae do not have an ortholog in M. uberis, suggesting that genome reduction was, at least in part, an independent process in each species. However, this observation does not exclude the possibility that the initial pseudogenization occurred in the ancestor of M. uberis and M. leprae. If this were the case, the pseudogenes that are present in both species would be more likely to share the same deleterious mutations, such as frameshifts and premature stop codons. We manually checked 50 random orthologous pseudogenes and identified only three pairs that shared one or more stop codons and/or frameshifts. Although some signals were lost or blurred by sequence drift, this observation suggests that some of the pseudogenization had already started in the ancestor of M. uberis and M. leprae and that the pseudogenization processes probably continued independently as the two species diverged. Note that roughly 500 genes which are missing in both species (compared to M. haemophilum) were likely deleted in the ancestor of M. uberis and M. leprae, which was probably adapting to a strict intracellular niche (6).

### Envelope biogenesis and other specific features.

M. leprae contains no methoxy-mycolates, probably because it has lost the MmaA2 and MmaA3 methoxy mycolic acid synthases (7). M. uberis has retained a functional MmaA3 (M. uberis BE_04680 [MUBE_04680]), which might influence the envelope composition. On the other hand, M. uberis has the same reduced set of five *mmpL* genes as M. leprae. It remains to be determined whether M. uberis produces a glycolipid similar or equivalent to the characteristic and highly antigenic phenolic glycolipid 1 of M. leprae.

A characteristic feature of M. leprae and M. lepromatosis is the presence of the horizontally acquired gene *proS*, encoding a eukaryote-like prolyl tRNA synthetase, which is both displaced and inverted with respect to the M. tuberculosis genome (7, 8) and is similar to those present in various members of the *Nocardiaceae* family. The same *proS* homolog is also present in M. uberis (MUBE_09850) and M. haemophilum (B586_RS07325) at the same genomic location, indicating that the gene was acquired by their ancestor. In addition, M. uberis has a cytochrome P450 (MUBE_02130) of unknown function that is similar to those present in more distantly related mycobacterial species but that is not present in M. leprae, M. lepromatosis, or M. haemophilum.

### Growth.

All attempts to grow M. uberis have failed, a result which was expected given its highly reduced genome. As in the cases of M. haemophilum and M. leprae, M. uberis lacks the mycobactin synthesis gene cluster present in Mycobacterium tuberculosis. Moreover, the gene coding for 50S ribosomal protein L25 (MUBE_04325) is truncated in M. uberis and is probably not functional. Disruption of this gene results in growth defects in M. tuberculosis (9) and Escherichia coli (10), due to reduced efficiency of the ribosome.

### Virulence.

The ESX-1 system is the main determinant of virulence in M. tuberculosis and in a number of other mycobacterial pathogens (11). While M. leprae and M. lepromatosis lost some components of ESX-1 (12), M. uberis retained the protein-coding capacity of the entire system (see Fig. S1 in the supplemental material). However, we identified a break in the genomic synteny downstream of *espJ* (*MUBE_00800*) and an insertion of a putative proline-proline-glutamate (PPE) gene (*MUBE_01185*), flanked by remnants of transposases, between *espB* (*MUBE_01195*) and *eccE_1_* (*MUBE_01180*). It is not clear how these changes impact the ESX-1 system in M. uberis. Curiously, the structural variations occur around the same genes that lost coding capacity in M. leprae and M. lepromatosis (Fig. S1).

10.1128/mSphere.00405-18.2 FIG S1 Genetic organization of the ESX-1 locus and the functionally associated *espACD* operon in M. uberis and comparison with M. tuberculosis, M. haemophilum, M. leprae, and M. lepromatosis. Colored arrows represent the various ESX genes encoding proteins belonging to different protein families (after Simeone et al.) (19). Empty arrows represent pseudogenes. Slashes separate distant genomic regions. Download FIG S1, EPS file, 0.1 MB. Copyright © 2018 Benjak et al. 2018 Benjak et al. This content is distributed under the terms of the Creative Commons Attribution 4.0 International license.

The ESX-5 system is the most recently evolved mycobacterial ESX system, which modulates virulence and host response, and is found only in the slow-growing mycobacterial species (13). The two *esx* genes and the flanking PE/PPE gene pair of the ESX-5 system underwent a series of duplication events that resulted in multiple copies scattered across the genome (14). It was shown that some of the paralog clusters in M. tuberculosis serve as accessory systems that aid in the secretion of a subset of proteins via the prototype ESX-5 system (14). Interestingly, M. uberis lost the core components of the prototype ESX-5 system, similarly to M. leprae, but has retained at least three paralog *esx* pairs.

### Drug susceptibility.

No mutations were found in the drug-resistance-conferring regions of RpoB (MUBE_04585), FolP1 (MUBE_01990), GyrA (MUBE_01070), and GyrB (MUBE_01075) (15), indicating that M. uberis is very likely susceptible to the antileprosy drugs rifampin, dapsone, and ofloxacin. Since there are no known molecular markers for resistance, we can only presume that M. uberis is also susceptible to the drugs clofazimine and clarithromycin, as is M. haemophilum (16).

### Mycobacterium leprae cluster organisms.

A recently reported causative agent of feline leprosy, “*Candidatus* Mycobacterium lepraefelis,” was found to be a close relative of M. leprae (17). The partial sequence of the *groEL2* (*hsp65*) gene from this pathogen is 89% to 90% identical to those of M. uberis, M. haemophilum, M. leprae, and M. lepromatosis. Phylogenetic reconstruction of the *groEL* sequence placed “*Candidatus* M. lepraefelis” between M. uberis and M. leprae (Fig. S2), so it is likely that the genomic structure of “*Candidatus* M. lepraefelis” resembles those of M. uberis and M. leprae. Efforts to close the genome sequences of the M. leprae-like pathogens are needed to facilitate more-detailed genomic comparisons, which, coupled with biological data, will provide further insights into the evolution and pathogenicity of this particular group of mycobacteria.

10.1128/mSphere.00405-18.3 FIG S2 Maximum likelihood phylogenetic tree of Mycobacterium uberis and selected mycobacterial species. The tree was created in MEGA7 based on the Tamura-Nei model from 372 nucleotide positions of the *groEL2* (*hsp65*) gene. The tree is drawn to scale, with branch lengths measured in the number of substitutions per site. Bootstrap support values, estimated from 500 replicates, are given below each branch. Download FIG S2, EPS file, 0.1 MB. Copyright © 2018 Benjak et al. 2018 Benjak et al. This content is distributed under the terms of the Creative Commons Attribution 4.0 International license.

### Significance and molecular detection of Mycobacterium uberis.

The availability of the genome sequence of M. uberis allowed us to design specific PCR primers for M. uberis. We confirmed the presence of the bacteria in the three cases of bovine nodular thelitis and the two cases of caprine nodular thelitis reported before (4, 5), as well as in a new case of bovine nodular thelitis and two cases of caprine nodular thelitis from different farms in France (Table 2). Moreover, we detected M. uberis in three cases of nodular tuberculoid scrotitis, confirming the implication of the bacterium in the two diseases. While definitive evidence is still lacking, these results strongly suggest that M. uberis is the causative agent of nodular thelitis and tuberculoid scrotitis.

**TABLE 2:** Samples used for the PCR detection of *M. uberis*^a^

Sample ID	Animal	Diagnosis	Tissue	Herd	Animal ID	Reference or source
13Z000257	Goat	NT	Mammary gland	A	goat 1	5
13Z002358	Goat	NT	Inguinal lymph node	A	goat 1	5
14Z002623	Goat	NT	Unspecified tissue	A	goat 2	5
14Z002624	Goat	NT	Mammary gland	A	goat 2	5
15Z001519	Goat	NT	Mammary gland	B	goat 3	This study
15Z001520	Goat	NT	Mammary gland	B	goat 4	This study
NA	Cow	NT	Mammary gland	C	cow 1	4
NA	Cow	NT	Mammary gland	C	cow 2	4
NA	Cow	NT	Mammary gland	D	cow 4	This study
NA	Bull	TS	Scrotum	E	taurus 1	This study
NA	Bull	TS	Scrotum	E	taurus 2	This study
NA	Bull	TS	Scrotum	E	taurus 3	This study

a ID, identifier; NT, nodular thelitis; TS, tuberculoid scrotitis; NA, not available.

Early detection and diagnosis of infectious diseases are crucial in animal husbandry to prevent disease outbreaks and contamination of animal products. Molecular tools from this study can be used for routine screening of the pathogen and will facilitate epidemiological investigations.

## MATERIALS AND METHODS

DNA was isolated from a skin biopsy of bovine udder with nodular thelitis and subjected to Illumina sequencing, followed by sequence assembly and annotation. More details are given in Text S1 in the supplemental material.

For the PCR assay, we used BLAST to identify genomic regions in M. uberis with no sequence homology to any publicly available sequence. We chose a 231-bp-long intergenic region that lies within the specific genomic island in the ESX-1 locus of M. uberis, between the *espB* gene and *eccE1* (Fig. S1), using primers Muber6F (5′-CACCGAACCCCTTCATGTCA-3′) and Muber6R (5′-CCCGGTAGTGTTGGCTTGAT-3′).

### Accession number(s).

The annotated genome has been deposited at DDBJ/ENA/GenBank under accession number QAYL00000000.
