# Supplementary material for: Highly Reduced Genome of the New Species Mycobacterium uberis, the Causative Agent of Nodular Thelitis and Tuberculoid Scrotitis in Livestock and a Close Relative of the Leprosy Bacilli
Source: mSphere. 2018 Oct 3;3(5):e00405-18. doi: 10.1128/mSphere.00405-18 (PMC6170788; doi:10.1128/mSphere.00405-18)
Supplement: TEXT S1 [file sph005182658s1.pdf]

The highly reduced genome of the new species *Mycobacterium uberis*, the causative agent of nodular thelitis and tuberculoid scrotitis in livestock, and a close relative of the leprosy bacilli

Andrej Benjak, Charlotte Avanzi, Yvonne Benito, Franck Breysse, Christophe Chartier, Maria-Laura Boschiroli, Christine Fourichon, Lorraine Michelet, Didier Pin, Jean-Pierre Flandrois, Pierre Bruyere, Oana Dumitrescu, Stewart T. Cole and Gerard Lina

SUPPLEMENTARY MATERIALS AND METHODS

DNA extraction. DNA was isolated from a skin biopsy of bovine udder with nodular thelitis. The diseased cow originated from the department of Jura in France. Skin tissues (50-200 mg) preserved in 70% ethanol were rehydrated in Hank's balanced solution (Thermo Fisher Scientific, USA, MA) prior to mincing with scissors. Cells were detached from the tissue by 30 min incubation at 37°C with a mixture of 0.5 U of collagenase and dispase (Roche Diagnostics, Mannheim, Germany), followed by incubation at 56°C with 10 mg/ml of trypsin (Sigma-Aldrich, St. Louis, Missouri, USA) until complete digestion. Free cells were resuspended in 1 mL phosphate-buffered saline (PBS) and DNA was extracted using the QIAmp DNA microbiome extraction kit (Qiagen, Hilden, Germany) according to the manufacturer's recommendations. Briefly, host cells were first lysed and host nucleic acids digested with a benzonase nuclease. Remaining bacterial cells were then lysed by chemical and mechanical disruption, followed by DNA precipitation and purification on silica column.

Illumina sequencing. DNA (50 µL) was sheared using the Covaris S220 Focused-ultrasonicator (Covaris) to obtain 400 bp-long DNA fragments, and purified using AMPure

beads (1.8x) following the manufacturer's protocol. The sheared DNA was quantified using the dsDNA High Sensitivity assay and a Qubit 2.0 fluorometer (Life Technologies). Up to 1 μg of DNA in 50 μL was used for library preparation using the Kapa Hyper prep kit (Roche) and PentAdapters (Pentabase) for indexing. The library was quantified using the dsDNA Broad Range assay and the Qubit 2.0 fluorometer. The library was sequenced on an Illumina HiSeq 2500 instrument (1 x 101 bp run).

Genome assembly. Illumina reads were assembled with MIRA v.4.9.5_2 (https://sourceforge.net/projects/mira-assembler/). The *de novo* sequence assembly resulted in 3,571 contigs larger than 1 kb and with an average coverage over 10x, amounting to 25.6 Mb of sequence.

It should be noted that assembling genomes from short reads has its limitations, especially in a metagenomic setting. For instance, repetitive sequences are probably largely missed in our analysis because these were likely assembled in very short contigs that do not resemble any known sequence. Similarly, the ribosomal genes were only partially recovered. Also, sporadic misassemblies cannot be excluded, as we spotted occasional inconsistencies between the contigs from the two assemblies. Nevertheless, sequence remains highly accurate (>99.9% match between orthologous contigs from the two assemblies, and no errors from mapping short reads back to the contigs), enabling a detailed view on the genic content of *M. uberis*.

Microbial composition. To estimate the fraction of *M. uberis* in our dataset, we analyzed the raw reads with MetaPhylerSR v.0.115 (1). The most abundant families were estimated to be Staphylococcaceae (81%), Micrococcaceae (12%), Mycobacteriaceae (4%) and Nocardiaceae (1%). Similarly, we assessed the microbial composition within the assembled sequence using taxator-tk v.1.3.1e (2), resulting in similar estimates (Staphylococcus 76%, Microbacteriaceae 5% and Mycobacterium 4%).

Identification of *M. uberis* sequence. Using discontiguous Mega BLAST, we identified 47 contigs that had extensive and continuous sequence similarity to the genome sequences of *Mycobacterium haemophilum* (GenBank CP011883.1), *Mycobacterium leprae* (GenBank AL450380.1) and *Mycobacterium lepromatosis* (GenBank JRPY01000000). The average coverage of the 47 contigs was 25.8x, and the GC content per contig ranged from 50.8% to 61.6% (average for all contigs = 57.5%). Contigs with a GC content below 40%, an average coverage above 50x and a length over 10 kb were discarded (we randomly checked several such contigs and they fully matched to other bacterial species). All remaining contigs larger than 1kb, including contigs that had sporadic short BLAST hits against the three genomes listed above, were then BLASTed against the non-redundant nucleotide sequences at NCBI, and confirmed to derive from other bacterial species.

Reads that did not map to the "unwanted" contigs (GC < 40%, coverage > 50x, length > 10 kb) were assembled with SPAdes v.3.5.0 (3), and the resulting contigs were analyzed as above. Seven additional contigs corresponding to *M. uberis* were identified, amounting to 119,835 nucleotides.

A total of 5.19% reads mapped to the draft genome sequence of *M. uberis*, which corresponds to the estimates from the taxonomic composition analysis described above.

Gene annotation. Genes were annotated with the NCBI Prokaryotic Genome Annotation Pipeline (PGAP) (https://www.ncbi.nlm.nih.gov/genome/annotation_prok/), followed by manual curation. PGAP predicted 2,212 protein-coding genes, and 747 pseudogenes. Nucleotide sequences of the predicted genes were compared to the genes from *M. haemophilum* (RefSeq NZ_CP011883.2) and *M. leprae* (https://mycobrowser.epfl.ch/) using discontiguous Mega BLAST. We manually checked all cases where multiple consecutive genes from *M. uberis* mapped to a single gene from *M. haemophilum* or *M. leprae*, as well as regions with short hypothetical genes, large intergenic regions, and regions with no homology

to *M. haemophilum* or *M. leprae*, by comparison with the protein database of NCBI using BLASTx. In most cases, erroneous predictions were due to underestimated or undetected pseudogenes.

***Mycobacterium uberis*-specific real-time PCR assay.** Real-time PCR was done on a LightCycler 2 instrument (Roche Diagnostics, Meyla, France) with 20 µl reaction mixtures containing 1X TaKaRa SYBR Premix Ex Taq™ II (Ozyme, Montigny-le-Bretonneux, France), 0,5 µM of each primer and 5 µL of DNA extract. A positive result was defined by the detection of a 231 bp amplicon with a melting temperature (Tm) of 85 ± 0.5°C.

Phylogenetic analysis. Protein sequences of ten proteins (DnaN, RplI, GrpE, MetG, RplY, PheT, FtsQ, HolA, MiaA, FtsY) (4) were aligned using ClustalW in BioEdit v. 7.1.3.0 (5) and concatenated from the following genome sequences: *M. uberis* Jura (this study), *M. leprae* TN (AL450380.1), *M. lepromatosis* Mx1-22A (JRPY01000000.1), *M. haemophilum* DSM 44634 ATCC 29548 (CP011883.2), *M. tuberculosis* H37Rv (NC_000962.3), *M. marinum* M (NC_010612.1), *M. ulcerans* Agy99 (CP000325.1), *M. avium* 104 (CP000479.1) and *M. abscessus* ATCC 19977 (NC_010397.1). Regions of poor alignment or containing extensive gaps were trimmed. The tree was created in MEGA7 (6) using the Maximum Likelihood method based on the JTT matrix-based model.

References

1. Liu B, Gibbons T, Ghodsi M, Treangen T, Pop M. 2011. Accurate and fast estimation of taxonomic profiles from metagenomic shotgun sequences. BMC Genomics 12 Suppl 2:S4.

2. Dröge J, Gregor I, McHardy AC. 2015. Taxator-tk: precise taxonomic assignment of metagenomes by fast approximation of evolutionary neighborhoods. Bioinformatics 31:817–824.

3. Bankevich A, Nurk S, Antipov D, Gurevich AA, Dvorkin M, Kulikov AS, Lesin VM, Nikolenko SI, Pham S, Prjibelski AD, Pyshkin AV, Sirotkin AV, Vyahhi N, Tesler G, Alekseyev MA, Pevzner PA. 2012. SPAdes: a new genome assembly algorithm and its applications to single-cell sequencing. J Comput Biol 19:455–477.

4. Mizuno T, Natori T, Kanazawa I, Eldesouky I, Fukunaga H, Ezaki T. 2016. Core housekeeping proteins useful for identification and classification of mycobacteria. Microb Resour Syst 32:25–37.

5. Hall T. 1999. BioEdit: a user-friendly biological sequence alignment editor and analysis program for Windows 95/98/NT. Nucleic Acids Symposium Series 41:95–98.

6. Kumar S, Stecher G, Tamura K. 2016. MEGA7: Molecular Evolutionary Genetics Analysis Version 7.0 for Bigger Datasets. Mol Biol Evol 33:1870–1874.
